# Supplementary material for: Prostaglandin in the ventromedial hypothalamus regulates peripheral glucose metabolism
Source: Nat Commun. 2021 Apr 20;12:2330. doi: 10.1038/s41467-021-22431-6 (PMC8058102; doi:10.1038/s41467-021-22431-6)
Supplement: Supplementary file 1 — Supplementary Information [file 41467_2021_22431_MOESM1_ESM.pdf]

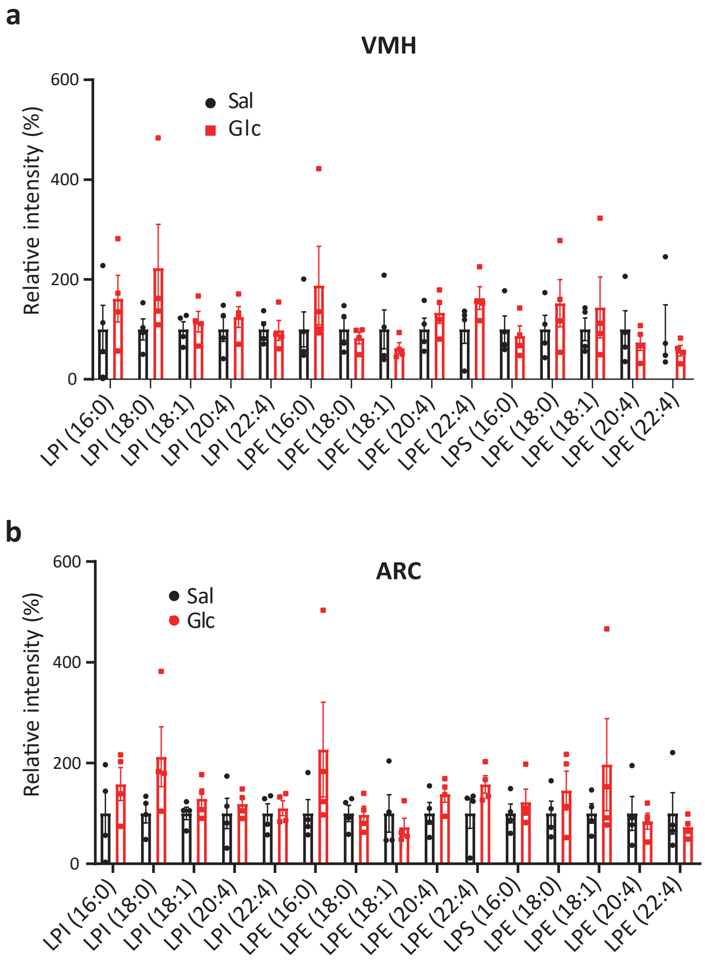

Supplementary Figure 1

Relative intensities of lysospecies in the VMH (a) and ARC (b) by IMS after injection with saline (n=4) or glucose (n=4).

All data represent the mean ± SEM. LPI: lysophosphatidylinositol, LPS: lysophosphatidylserine, LPE: lysophosphatidylethanolamine.

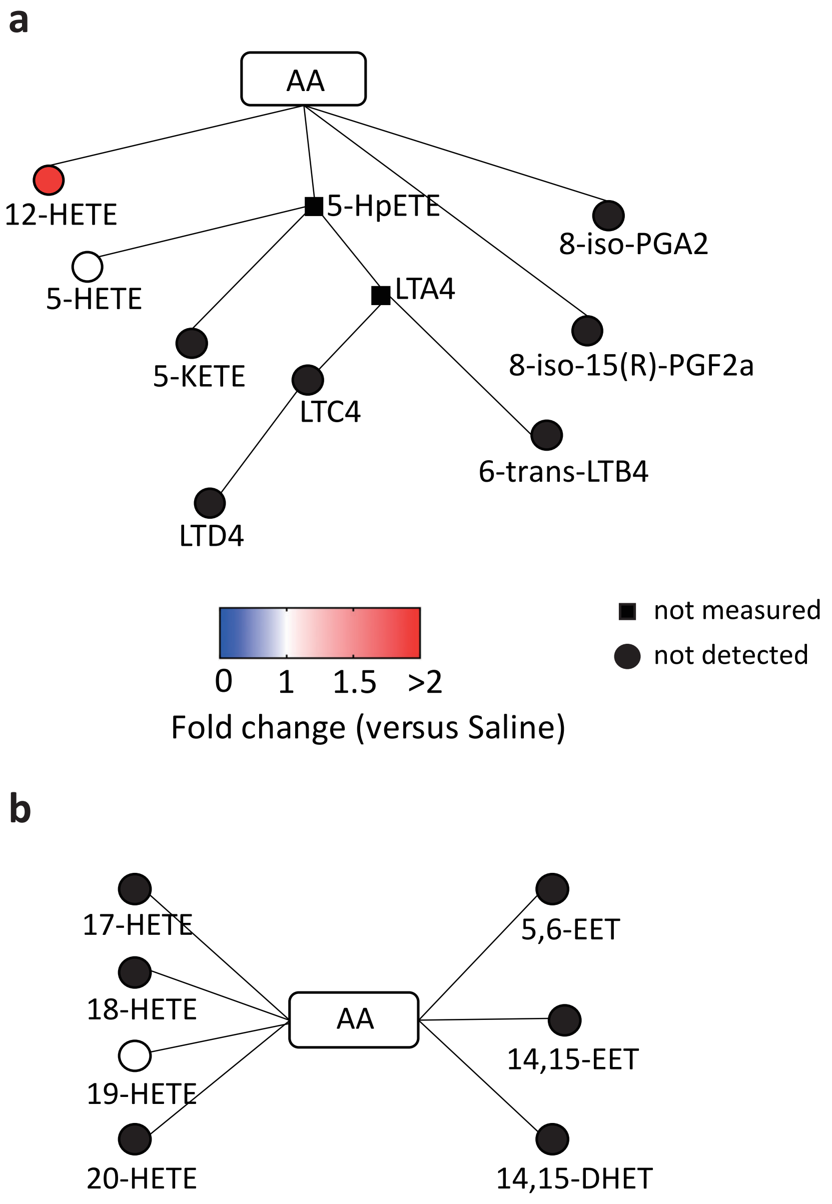

Supplementary Figure 2
Relative amounts of hypothalamic eicosanoids mediated by lipoxygenase (a) or cytochrome P450 (b) after the injection of glucose compared with saline injected mice. n=3 in each experimental group. Data represent the mean fold change in color.

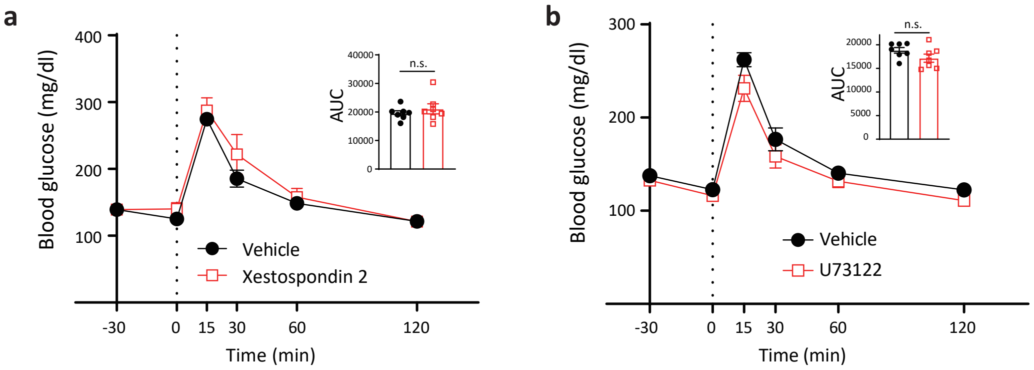

Supplementary Figure 3
Hypothalamic PLC-mediated-pathway does not affect systemic glucose metabolism. (a) Glucose tolerance test (GTT) (0–120 min) after intra-hypothalamic injection (-30 min) of Xestospondin, an IP3 receptor antagonist, (n=7) or vehicle (n=7). (b) GTT (0–120 min) after intra-hypothalamic injection (-30 min) of U73122, a phospholipase C (PLC) inhibitor, (n=7) or vehicle (n=7). All data represent the mean ± SEM

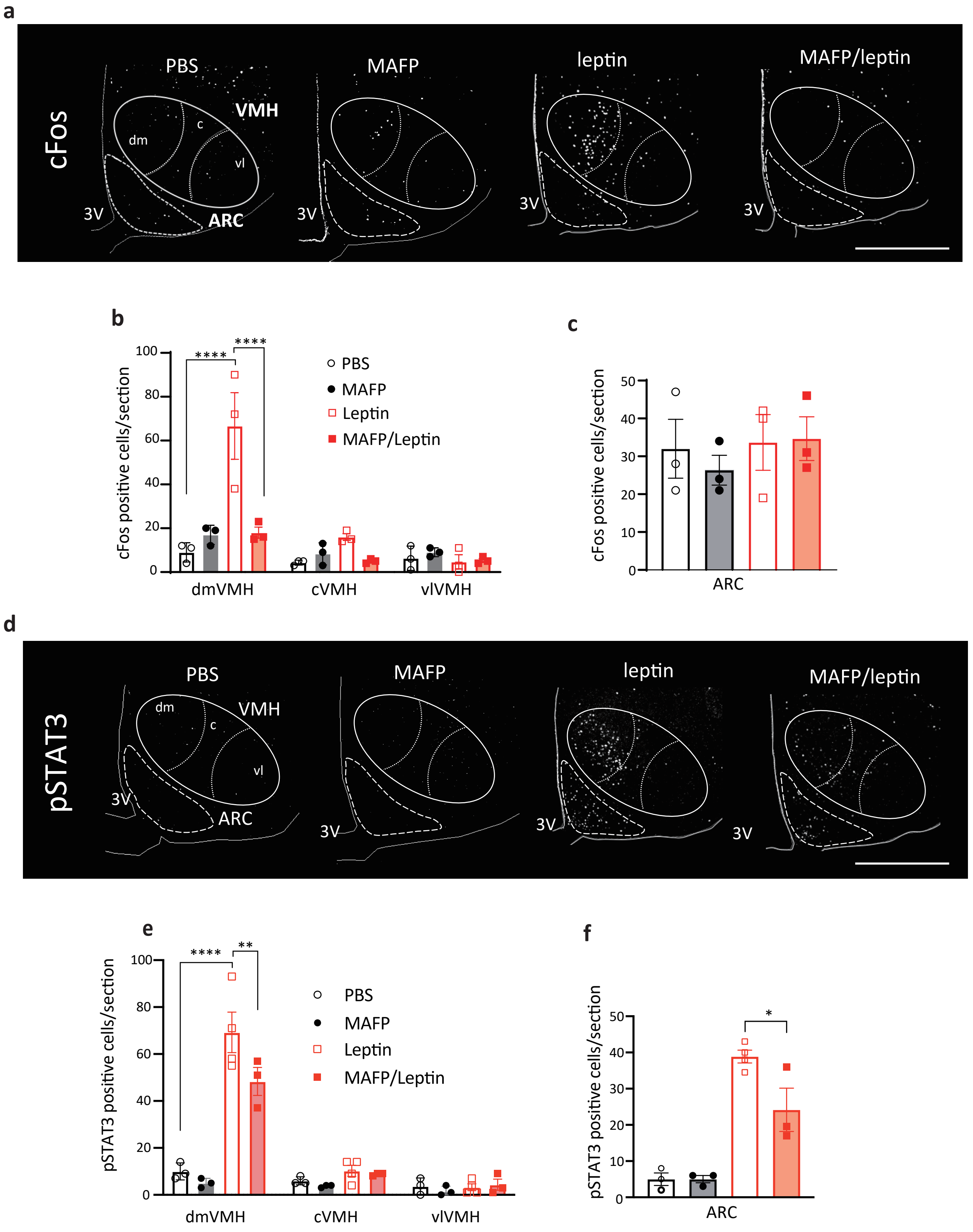

Supplementary Figure 4
Hypothalamic leptin responsiveness is regulated by cPLA2.
a, Representative micrographs showing immunofluorescent cFos staining in the hypothalamus of mice i.c.v. injected with PBS, MAFP, leptin or both MAFP and leptin. Scale bar: 500 µm. b, c, Quantification of cFos expression in the dmVMH, cVMH, vlVMH (b), and ARC (c) after i.c.v. injection of PBS, MAFP, leptin or both MAFP and leptin (n=3 in each experimental group) (VMH: two-way ANOVA followed by Sidak multiple comparison test, in b, $p < 0.0001$ PBS vs Leptin, $p < 0.0001$ Leptin vs MAFP/Leptin.). d, Representative micrographs showing immunofluorescent pSTAT (Tyr 705) staining in the hypothalamus of mice i.c.v. injected with PBS, MAFP, leptin or both MAFP and leptin. Scale bar: 500 µm. e, f, Quantification of pSTAT3 (Tyr 705)-positive cells in the dmVMH, cVMH, vlVMH (e), and ARC (f) after i.c.v. injection of PBS, MAFP, leptin or both MAFP and leptin (n=4 in each experimental group) (VMH: two-way ANOVA followed by Sidak multiple comparison test, in e, $p < 0.0001$ Leptin vs PBS, p= 0,0095 Leptin vs MAFP/Leptin. In f, one-way ANOVA followed by Sidak multiple comparison test, p = 0.0408 Leptin vs MAFP/Leptin.).
All data represent the mean ± SEM; * = p<0.05; ** = p<0.01; *** = p<0.001; **** = p<0.0001.

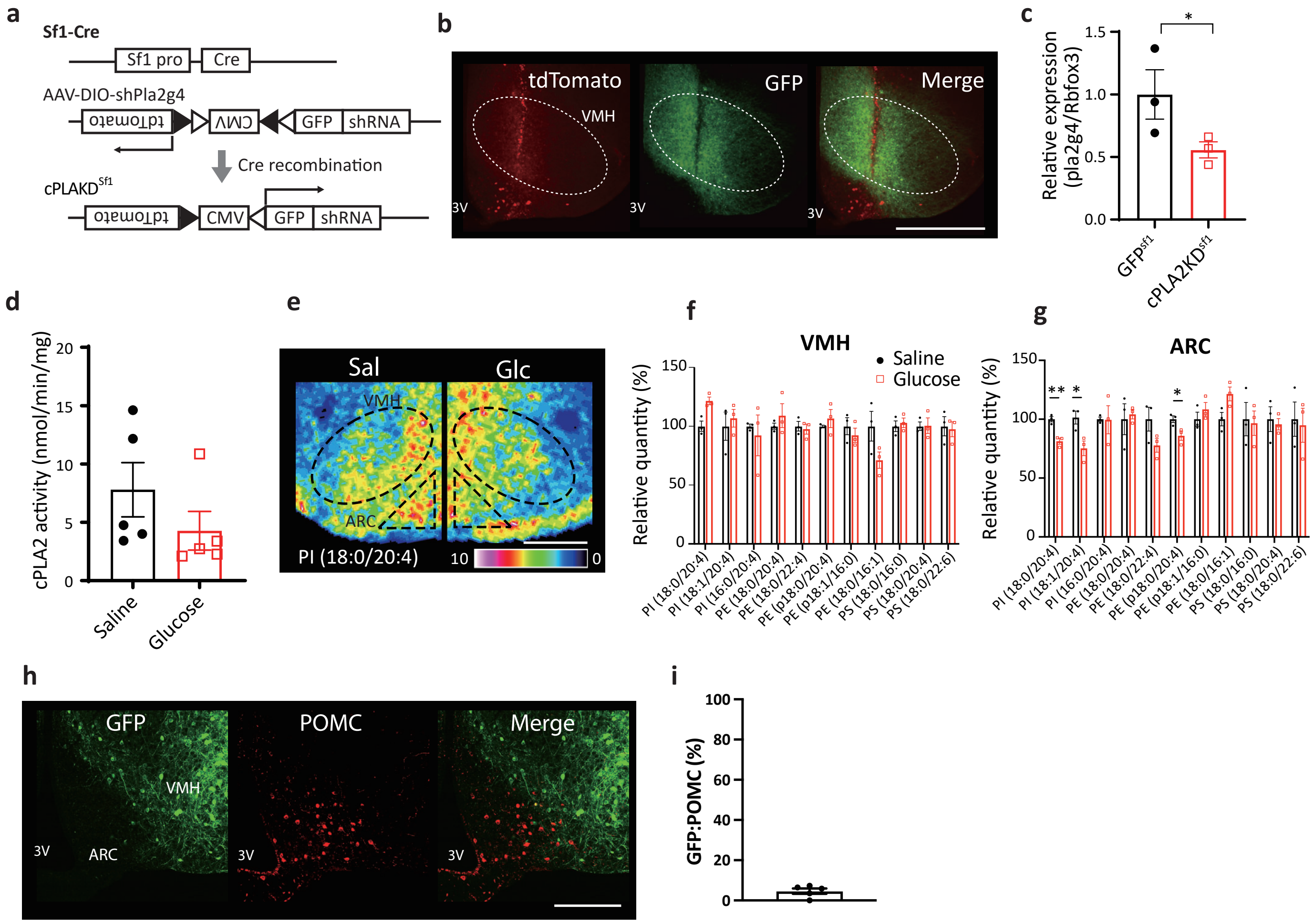

Supplementary Figure 5
a, Construct of AAV8-DIO (CreOn)-shRNA against *mpla2g4*, containing DIO (Double-floxed Inverted Open reading frame) to express shRNA Cre-dependently. b, Representative micrographs showing virus infected (tdTomato) and shRNA expressing (GFP) Sf1-neurons. Scale bar: 500 μm. c, Expression of pla2g4a mRNA in the whole hypothalamus injected with AAV8-DIO-shRNA against mpla2g4 (cPLA2KD^{Sf1}; n=3) compared with control mice (GFP^{Sf1}; n=3) (two-tailed t test, p = 0.0497, cPLA2KD^{sf1} vs GFP^{sf1}). d, Enzymatic activity of cPLA2 after injection of saline (n=5) or glucose (2g/kg) (n=5) in C57BL/6J mice. e-g, Distributions of phospholipids in the hypothalamus of cPLA2KD^{Sf1} mice after i.p. injection of saline or glucose. e, Representative results of IMS on hypothalamic phosphatidylinositol (PI) (18:0/20:4) of cPLA2KD^{Sf1} mice after saline (left half) or glucose injection (right half). Scale bar: 500 μm. f, g, Relative intensities of phospholipids in the VMH (f) and ARC (g) of cPLA2KD^{Sf1} mice after injection of saline (n=3) or glucose (n=3). (two-tailed t test for each molecule, in g, p = 0.0056 for PI (18:0/20:4), p = 0.0343 for PI (18:1/20:4), p = 0.0453 for PE (p18:0/20:4)). h, Representative micrographs showing immunofluorescence of POMC (α-MSH) and GFP in Sf1-cre mice injected with AAV-DIO-GFP. Scale bar: 200 μm. i, Ratio of GFP expressed POMC neurons (n=5). All data represent the mean ± SEM; * = p<0.05; ** = p<0.01

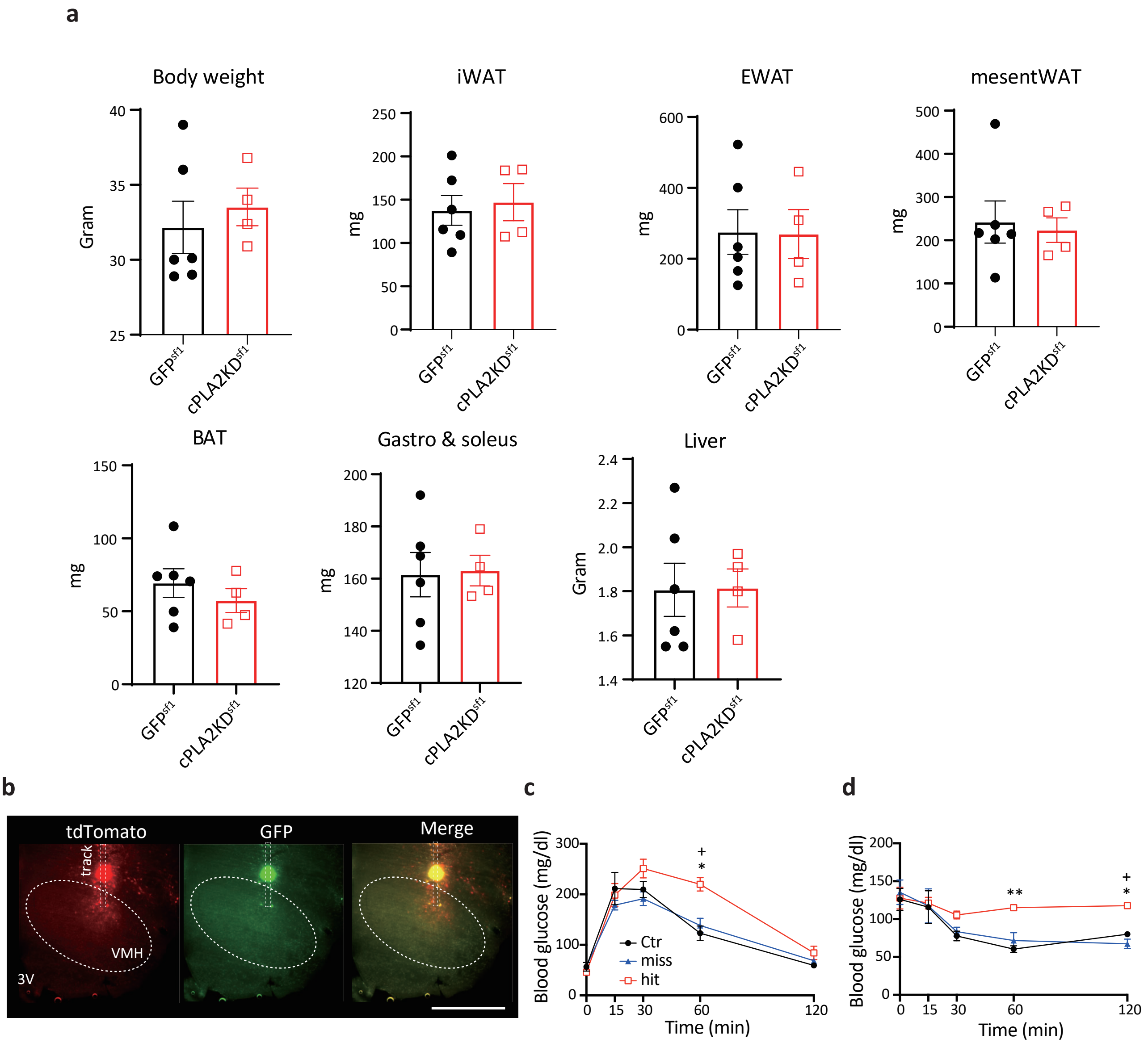

Supplementary Figure 6

a, Body weight and tissue weight in cPLA2KD^{Sf1} mice (n=4) and GFP^{Sf1} mice (n=5) 8 weeks after the AAV injection. (iWAT: inguinal white adipose tissue. EWAT: epididymal white adipose tissue. mesentWAT: mesenteric white adipose tissue. BAT: brown adipose tissue). b-d, Phenotype of mice with “hit” and “miss” injection of AAV8-DIO-shRNA against *mpla2g4* into the VMH in Sf1-cre mice. b, Representative micrographs showing the “miss” injection of virus into the VMH. c, Glucose tolerance test on the Sf1-cre mice received “miss” (n=3) or “hit” (n=4) injection of AAV8-DIO-shRNA against *mpla2g4* or hit injection of AAV-DIO-GFP (Ctr) (n=3) (two-way ANOVA followed by Sidak multiple comparison test, p = 0.0156 at time = 60 Ctr vs hit, p = 0.0302 at time =60 miss vs hit). d, Insulin tolerance test on the mice receive “miss” (n=3) or “hit” (n=3) injection or hit injection of AAV-DIO-GFP (Ctr) (n=3) (two-way ANOVA followed by Sidak multiple comparison test, p = 0.0022 at time = 60 Ctr vs hit, p = 0.0117 at time = 120 Ctr vs hit, p = 0.0141 at time = 120, miss vs hit). Data represent the mean ± SEM. * = p<0.05 (Ctr versus hit) ; ** = p<0.01 (Ctr versus hit); + = p<0.05 (hit versus miss).

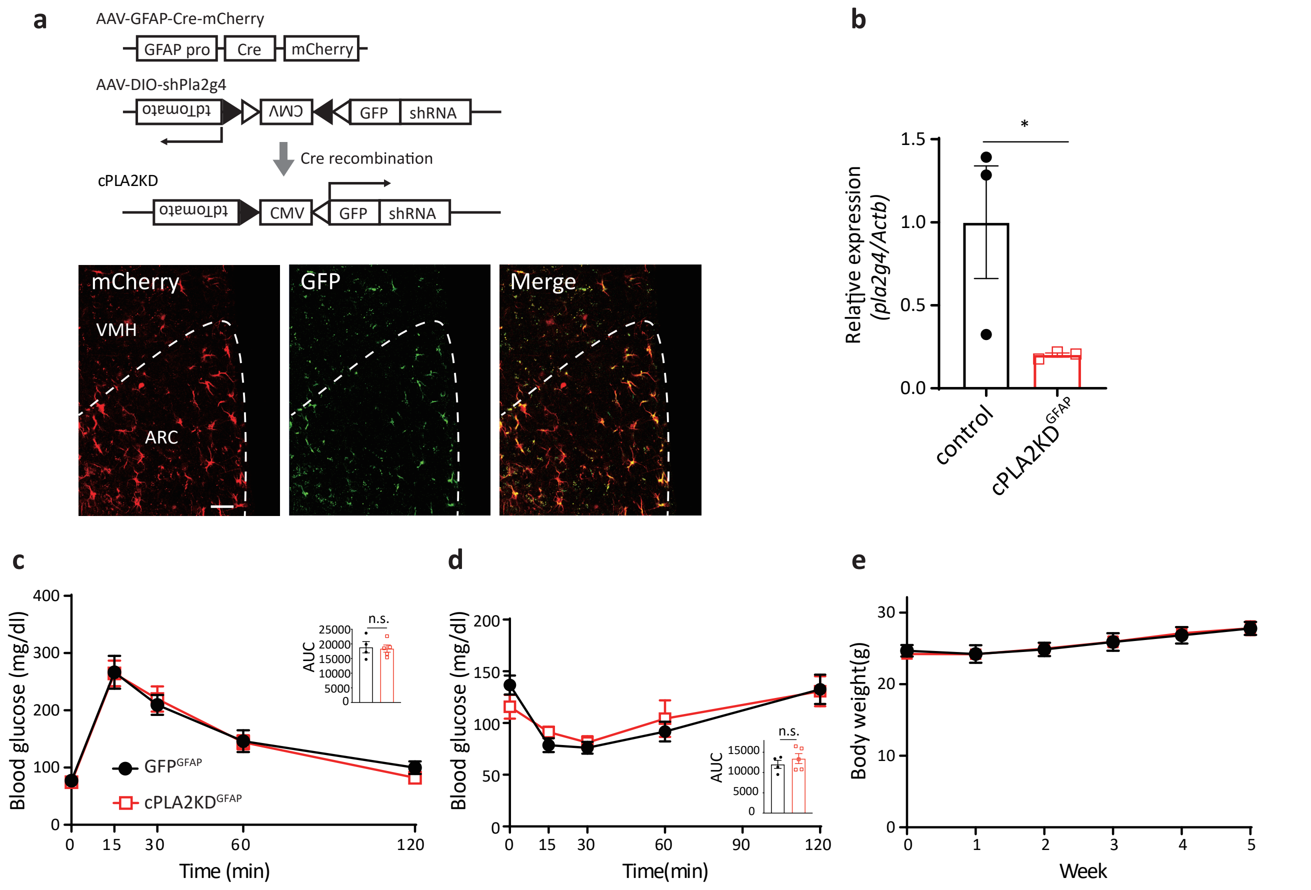

Supplementary Figure 7

Knockdown of astrocytic cPLA2 in hypothalamus (cPLA2KD^{GFAP}) did not change body weight and glucose metabolism.

a, Construct of AAV8-GFAP-Cre-mCherry and AAV8-DIO-shRNA against *mpla2g4* and representative micrographs showing virus infected (tdTomato) and shRNA expressing (GFP) astrocytes in the ARC. Scale bar: 25 μm. b, Relative expression of cPLA2 in the hypothalamus of cPLA2KD^{GFAP} and control mice. n=3 in each experimental group (two-tailed t test, p = 0.039, Ctr vs cPLA2KD^{GFAP}). c, Glucose tolerance test in cPLA2KD^{GFAP} mice (n=5) and GFP^{GFAP} mice (n=4). d, Insulin tolerance test in cPLA2KD^{GFAP} mice (n=5) and GFP^{GFAP} mice (n=4). e, Body weight change in cPLA2KD^{GFAP} mice (n=5) and GFP^{GFAP} mice (n=4) after viral injection. All data represent the mean ± SEM. * = p<0.05

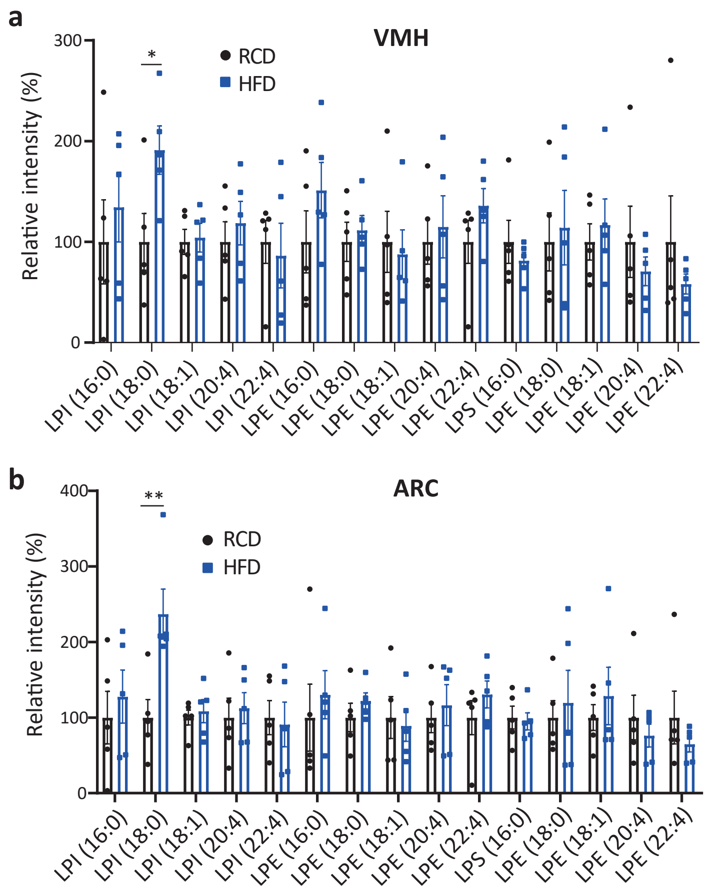

Supplementary Figure 8

Relative intensities of lypospecies in the VMH (a) and ARC (b) by IMS from RCD (n=5) or HFD(n=5) fed mice. (two-tailed t test for each molecule, LPI(18:0) in VMH, p = 0.0389 RCD vs HFD, in ARC p=0.0099 RCD vs HFD).

All data represent the mean ± SEM; * = p<0.05; ** = p<0.01.

LPI: lysophosphatidylinositol, LPS: lysophosphatidylserine, LPE: lysophosphatidylethanolamine.

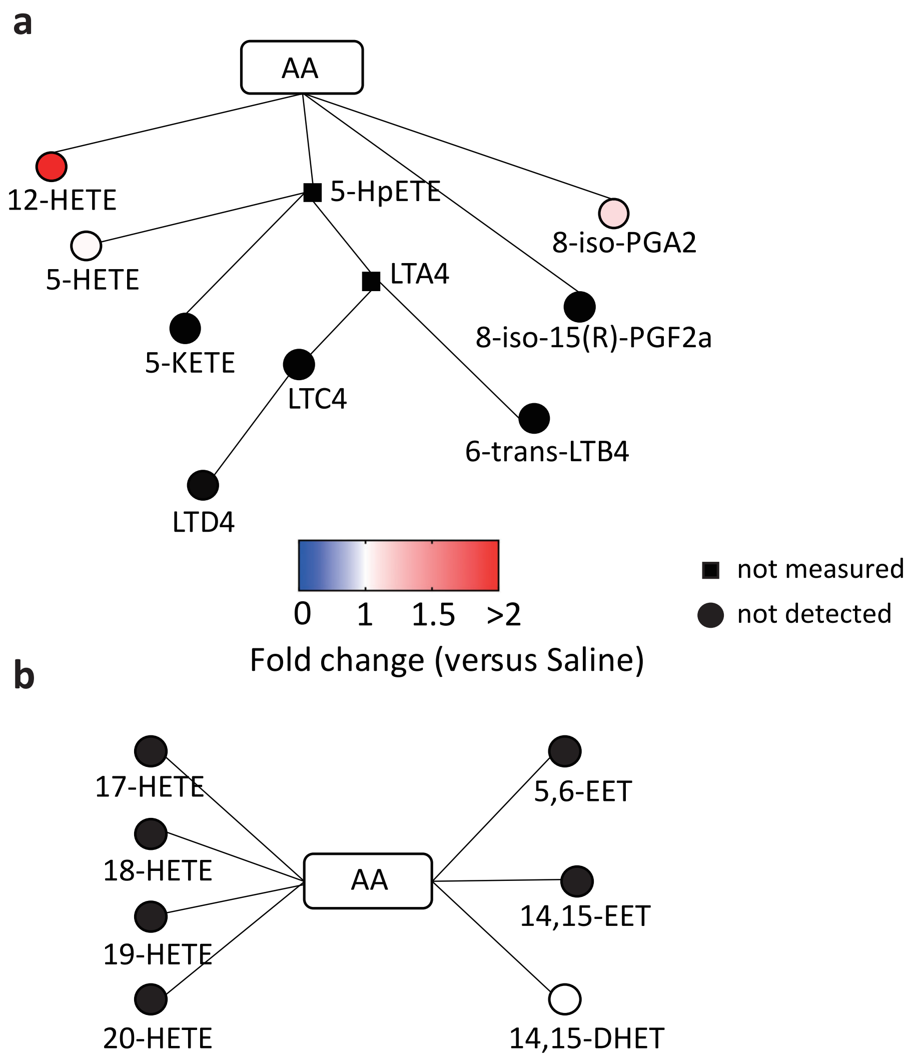

Supplementary Figure 9
Relative amounts of hypothalamic eicosanoids mediated by lipoxygenase (**a**) or cytochrome P450 (**b**) in RCD or HFD fed mice. n=3 each experimental group. Data represent the mean fold change in color.

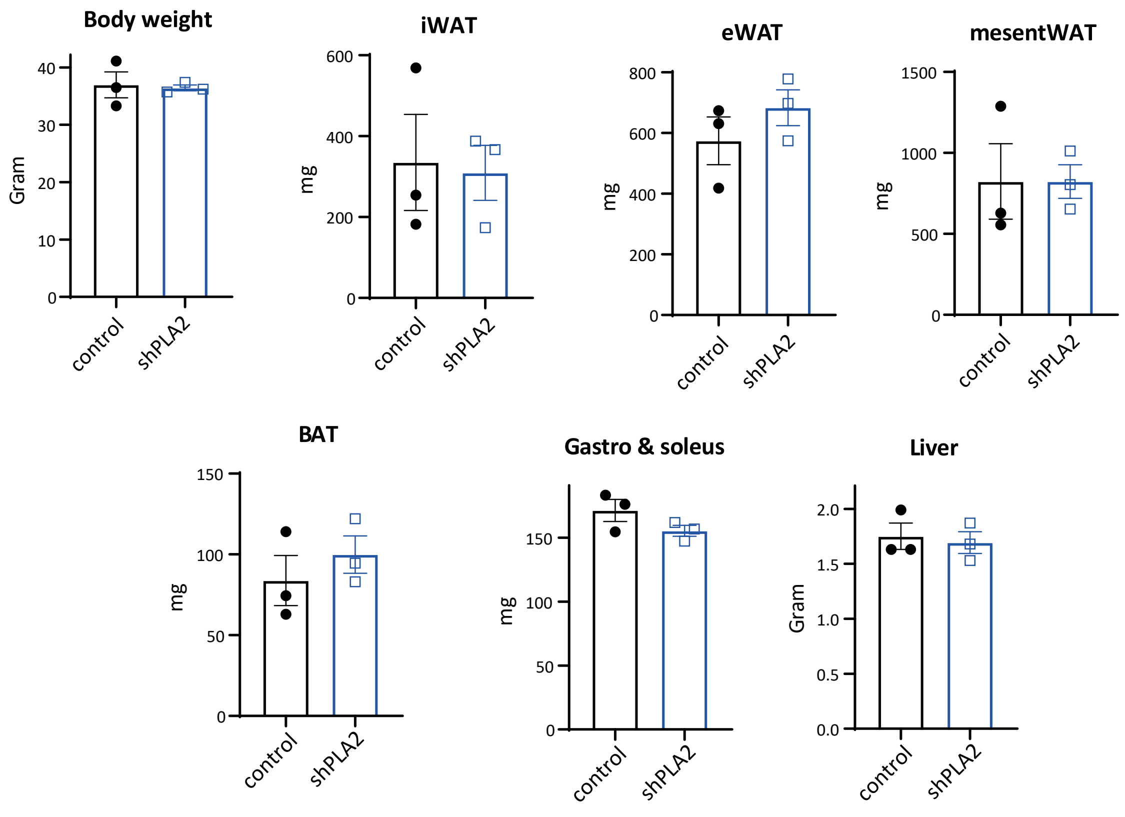

Supplementary Figure 10
Body weight and tissue weight in male cPLA2KD^{Sf1} mice (n=3) and GFP^{Sf1} mice (n=3) after 8 weeks of a HFD feeding. (iWAT: inguinal white adipose tissue. EWAT: epididymal white adipose tissue. mesentWAT: mesenteric white adipose tissue. BAT: brown adipose tissue). All data represent the mean ± SEM.

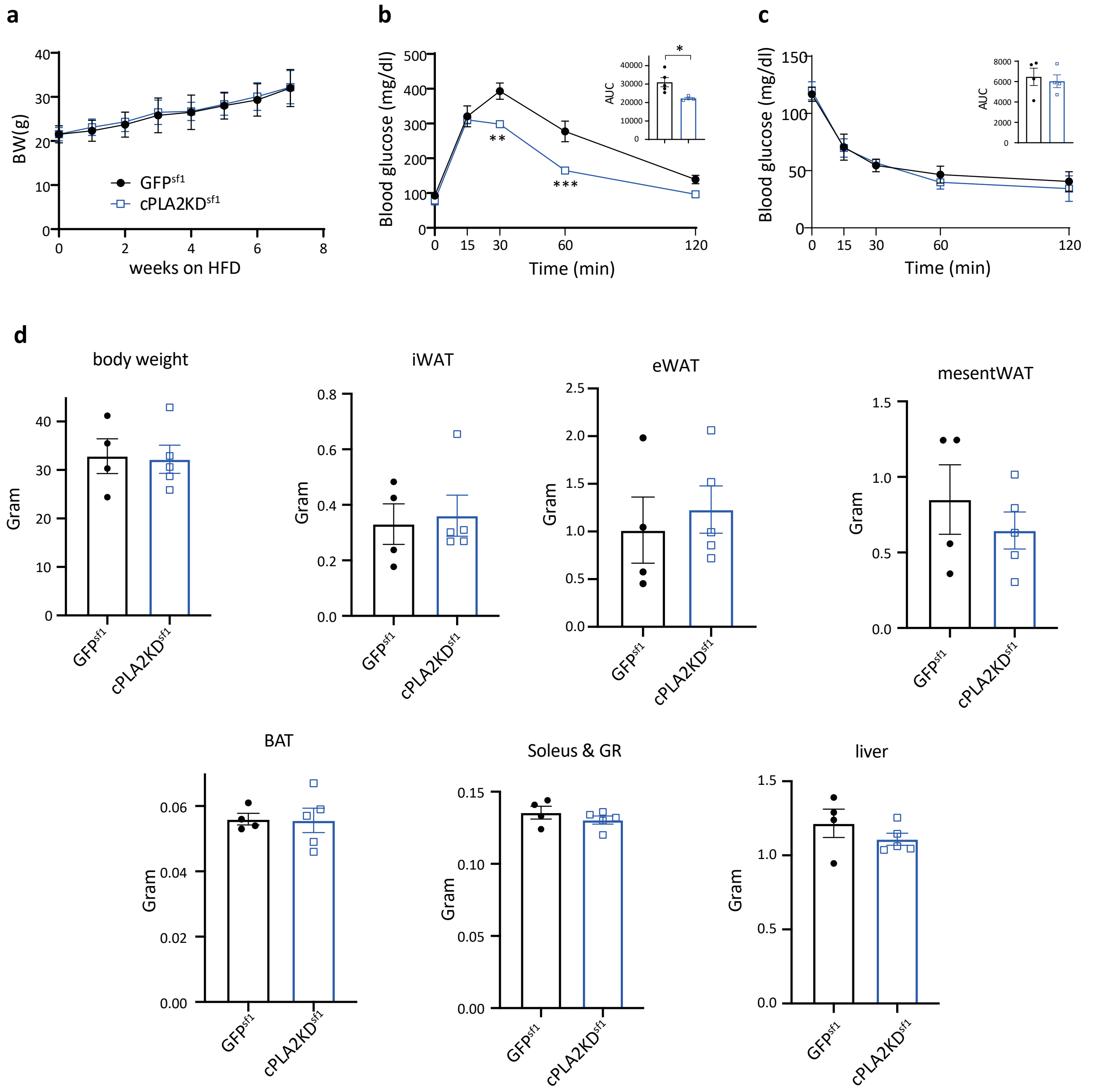

Supplementary Figure 11

Knockdown of cPLA2 improves HFD-induced impairment of glucose metabolism in female mice. a, Body weight change in female cPLA2KD^{Sf1} mice (n=4) and GFP^{Sf1} mice (n=4) under HFD. b, Glucose tolerance test on female cPLA2KD^{Sf1} mice (n=4) and GFP^{Sf1} mice (n=5). (two-way ANOVA followed by Sidak multiple comparison test, p = 0.0059 at time = 30, p = 0.0009 at time = 60, GFP^{Sf1} vs cPLA2KD^{Sf1}. two-tailed t test in area under the curve (AUC) during GTT, p = 0.0177, GFP^{Sf1} vs cPLA2KD^{Sf1}). c, Insulin tolerance test on cPLA2KD^{Sf1} (n=4) mice and GFP^{Sf1} mice (n=4) after 8 weeks of HFD feeding. d, Body weight and tissue weight in cPLA2KD^{Sf1} mice (n=5) and GFP^{Sf1} mice (n=4) after 8 weeks of HFD feeding. (iWAT: inguinal white adipose tissue. EWAT: epididymal white adipose tissue. mesentWAT: mesenteric white adipose tissue. BAT: brown adipose tissue.) All data represent the mean ± SEM; * = p<0.05; ** = p<0.01; *** = p<0.001.

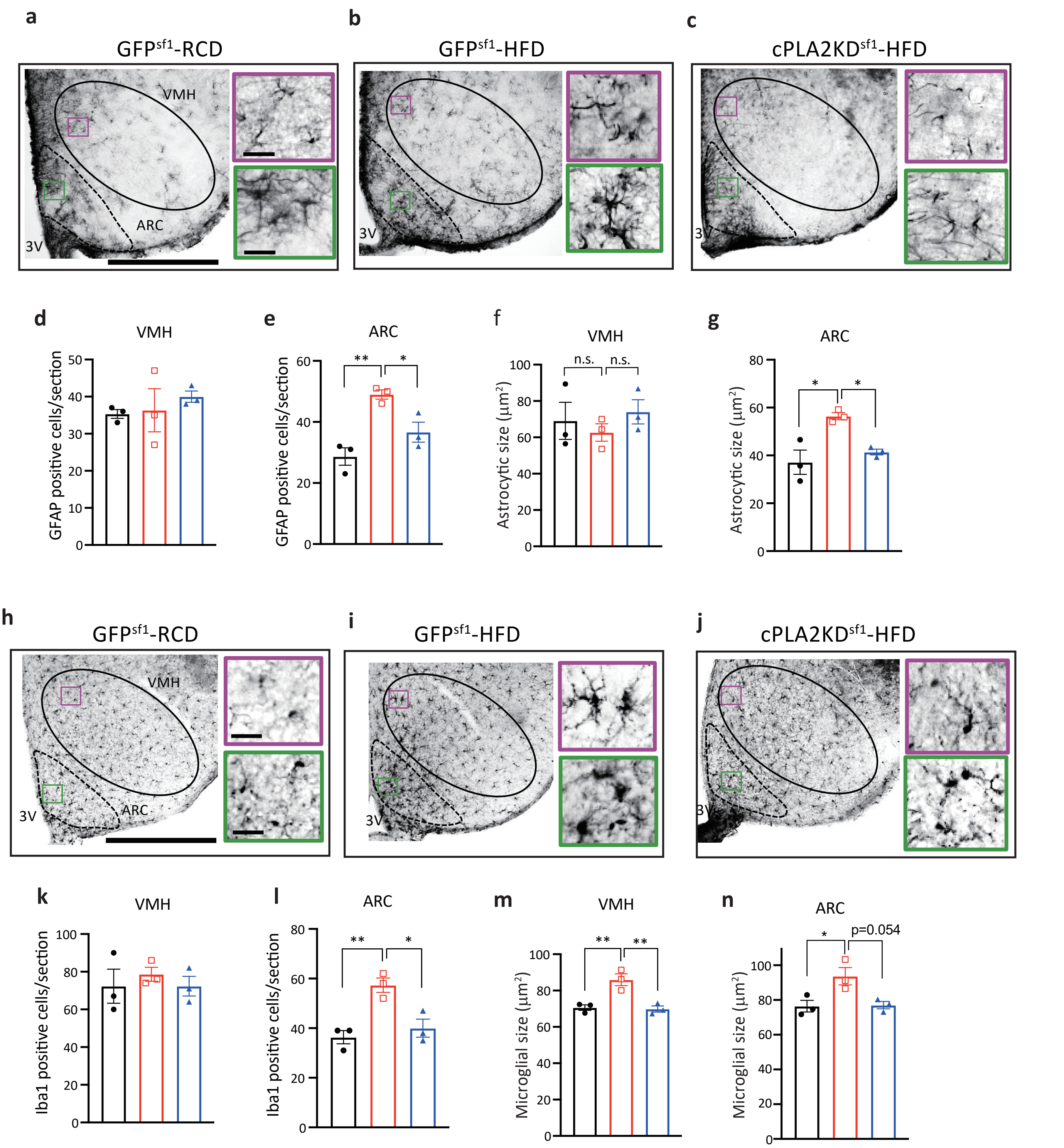

Supplementary Figure 12

Knockdown of cPLA2 prevents HFD-induced microgliosis and astrogliosis in female mice.

a-c, Left: Representative micrographs showing immunofluorescent GFAP staining in the hypothalamus of RCD-fed GFP^{sf1} mice (GFP^{sf1}-RCD) (a), HFD-fed GFP^{sf1} mice (GFP^{sf1}-HFD) (b) and HFD-fed $cPLA2KD^{sf1}$ ($cPLA2KD^{sf1}$-HFD) mice (c). Scale bar: 500 µm. Right: magnified rectangles showed in left. Scale bar: 30 µm. d,e, Quantification of GFAP-positive cells in the VMH (d) or ARC (e) of GFP^{sf1}-RCD (n=3), GFP^{sf1}-HFD (n=3) and $cPLA2KD^{sf1}$-HFD (n=3) mice (one-way ANOVA followed by Sidak multiple comparison test, in e, p = 0.005 GFP^{sf1} RCD vs GFP^{sf1} HFD, p= 0.0497 GFP^{sf1} HFD vs $cPLA2KD^{sf1}$ HFD). f,g, Size of GFAP positive cells in in the VMH (f) or ARC (g) of GFP^{sf1}-RCD (n=3), GFP^{sf1}-HFD (n=3) and $cPLA2KD^{sf1}$-HFD (n=3) mice (one-way ANOVA followed by Sidak multiple comparison test, in g, p = 0.0004 GFP^{sf1} RCD vs GFP^{sf1} HFD, p= 0.0128 GFP^{sf1} HFD vs $cPLA2KD^{sf1}$ HFD). h-j, Left: Representative micrographs showing immunochemistry Iba1 staining in the hypothalamus of GFP^{sf1}-RCD (h), GFP^{sf1}-HFD (i) and $cPLA2KD^{sf1}$-HFD mice (j). Scale bar: 500 µm. Right: magnified rectangles showed in left. Scale bar: 30 µm. k,l, Quantification of Iba1-positive cells in the VMH (k) or ARC (l) of GFP^{sf1}-RCD (n=3), GFP^{sf1}-HFD (n=3) and $cPLA2KD^{sf1}$-HFD (n=3) mice (one-way ANOVA followed by Sidak multiple comparison test, in l, p = 0.0089 GFP^{sf1} RCD vs GFP^{sf1} HFD, p= 0.0219 GFP^{sf1} HFD vs $cPLA2KD^{sf1}$ HFD). m,n, Size of Iba1 positive cells in in the VMH (m) or ARC (n) of GFP^{sf1}-RCD (n= 3), GFP^{sf1}-HFD (n=3) and $cPLA2KD^{sf1}$-HFD (n=3) mice (one-way ANOVA followed by Sidak multiple comparison test, in m, p = 0.0162 GFP^{sf1} RCD vs GFP^{sf1} HFD, p= 0.0077 GFP^{sf1} HFD vs $cPLA2KD^{sf1}$ HFD, in n, p = 0.0042 GFP^{sf1} RCD vs GFP^{sf1} HFD, p= 0.0038 GFP^{sf1} HFD vs $cPLA2KD^{sf1}$ HFD). All data represent the mean ± SEM; * = $p<0.05$; ** = $p<0.01$.

Supplementary table 1 List of reagents and resources

REAGENT or RESOURCE	SOURCE	IDENTIFIER
Antibodies		
Rabbit-anti-cFos	Santa Cruz	CAT#SC-52
Rabbit-anti-Iba1	FUJIFILM Wako	CAT#LKN5648
Rabbit-anti-GFAP	Sigma-Aldrich	CAT#HPA056030
Rabbit-anti-GFP	Frontier institute	AB_2571573
Rabbit-anti-pSTAT3 (Tyr 705) antibody	Cell Signaling Technologies	9145S
Phospho-cPLA2 (Ser505) Antibody	Cell Signaling Technologies	2831S
cPLA2 Antibody	Cell Signaling Technologies	2832S
Streptavidin, DyLight 488 Conjugated	Vector Biolabs	SA-5488
Biotinylated-Goat-Anti-Rabbit (IgG) secondary antibody	Thermo Fisher Scientific	A11034
Anti-rabbit IgG (H+L), F(ab')2 Fragment (Alexa Fluor 647 Conjugate)	Cell Signaling Technologies	4414S
Anti-rabbit IgG (H+L), F(ab')2 Fragment (Alexa Fluor® 488 Conjugate)	Cell Signaling Technologies	4412S
Bacterial and Virus Strains		
AAV8-DIO (Cre-On)-shRNA against *mpla2g4*	Vigene	Custom made
AAV8-GFAP-mcherry-Cre	UNC Vector Core	Lot# AV5056C
Chemicals, Peptides, and Recombinant Proteins		
9-Aminoacridine hemihydrate	Thermo Fisher Scientific	134410010
Glucose	FUJIFILM Wako	049-31165
Novolin R 100 IU	Novo Nordisk	N/A
Glucose-D-[3-^{3}H]	Muromachi Kikai	ART0124
2-Deoxy-D-glucose ^{14}C(U)	Muromachi Kikai	ARC0112A
Methyl arachidonyl fluorophosphonate (MAFP)	Sigma-Aldrich	M2939
Indomethacin (Indo)	Sigma-Aldrich	I7378
Ethanol	FUJIFILM Wako	056-03341
Dimethyl sulfoxide	Nacalai Tesque	13407
TRIzol™ reagent	Thermo Fisher Scientific	15596026
Critical Commercial Assays		
Mouse Insulin ELISA KIT	FUJIFILM Wako	633-23919
Cytosolic Phospholipase A2 Assay Kit	Abcam	Ab133090
Secretory-phospholipase-A2 Assay Kit	Abcam	Ab133089
TaqMan™ Gene Expression Master Mix	Thermo Fisher Scientific	4369016
M-MLV Reverse Transcriptase	Thermo Fisher Scientific	28025013
VECTASTAIN Elite ABC Kit	Vector Laboratories	PK-6100
DAB tablet	FUJIFILM Wako	045-22833

Supplementary table 2

Assignment of lipid molecular species by IMS negative ion mode

Lipid assignment	[M-H]$^-$(*m/z*)
Palmitic acid	255.25
Oleic acid	281.3
Stearic acid	283.35
Arachidonic acid	303.3
DHA	327.33
PE (p18:1/16:0), plasmalogen	700.6
PE (18:0/16:1)	716.6
PE (p18:0/20:4), plasmalogen	750.5
PS (18:0/16:0)	762.6
PE (18:0/20:4)	766.5
PE (18:0/22:4)	794.5
PS (18:0/20:4)	810.6
PS (18:0/22:6)	834.6
PI (16:0/20:4)	857.6
PI (18:1/20:4)	883.55
PI (18:0/20:4)	885.77
LPE 16:0	452.3
LPE 18:0	480.3
LPE 18:1	478.3
LPE 20:4	500.3
LPE 22:4	528.3
LPI 16:0	571.3
LPI 18:0	599.3
LPI 18:1	597.3
LPI 20:4	619.3
LPI 22:4	647.3
LPS 16:0	496.3
LPS 18:0	524.3
LPS 18:1	522.3
LPS 20:4	544.3
LPS 22:4	572.3

Supplementary table 3

MRM conditions for each PGs

Compoubds	Retention time (min)	SRM		SRM (validation)		Polarity	Collision energy (eV)
		Q1	Q3	Q1	Q3		
tetranor-PGFM	2.7	329.2	311.2			Negative	15
tetranor-PGEM-d6	3.4	333.2	315.2			Negative	12
tetranor-PGEM	3.1	327.2	309.2			Negative	15
tetranor-PGDM	3.2	327.2	309.2			Negative	15
20-hydroxy-PGF2a	5.5	369.2	325.2			Negative	21
20-hydroxy-PGE2	5.9	367.2	287.2			Negative	18
18-carboxy-dinor-LTB4	6.2	337.2	59.1			Negative	26
13,14-dihydro-15-keto-tetranor-PGF1b	6.7	299.2	113.1			Negative	24
2,3-dinor-8-iso-PGF2a	7.3	325.2	237.2			Negative	13
13,14-dihydro-15-keto-tetranor-PGF1a	7.6	299.2	113.1			Negative	24
6-keto-PGF1a-d4	7.6	373.2	249.1			Negative	27
6-keto-PGF1a	7.7	369.2	245.2			Negative	27
13,14-dihydro-15-keto-tetranor-PGD2	8.0	297.2	109.1			Negative	23
20-carboxy-LTB4	8.2	365.2	169.1			Negative	22
PGF2a-EA	7.8	442.3	334.2			Negative	22
PGE2-EA	8.3	440.3	271.2			Negative	26
8-iso-PGF3a	8.3	351.2	307.2			Negative	18
TXB3	8.5	367.2	169.1			Negative	19
20-hydroxy-LTB4	8.4	351.2	195.1			Negative	18
PGE1-EA	8.5	442.3	360.2			Negative	15
13,14-dihydro-15-keto-tetranor-PGE2	8.4	297.2	109.1			Negative	21
PGD2-EA	8.7	440.3	271.2			Negative	25
6,15-diketo-13,14-dihydro-PGF1a	8.9	369.2	113.1			Negative	31
PGF3a	9.3	351.2	193.1			Negative	25
iPF2a-IV	9.6	353.2	127.1			Negative	24
8-iso-15(R)-PGF2a	9.4	353.2	193.1			Negative	27
TXB1	9.7	371.2	171.2			Negative	20
8-iso-PGF2a	9.5	353.2	193.1			Negative	27
TXB2-d4	9.6	373.2	199.1			Negative	15
TXB2	9.4	369.2	195.1			Negative	14
PGE3	9.4	349.2	269.2			Negative	15
8-iso-PGF1a	9.7	355.2	293.2			Negative	24
11-beta-PGF2a	9.8	353.2	193.1			Negative	25
PGD3	9.9	349.2	269.2			Negative	15
5-iPF2a-VI	10.0	353.2	115.1			Negative	21
8-iso-15-keto-PGF2a	10.1	351.2	219.2			Negative	17
PGF2a-d4	10.2	357.2	197.2			Negative	26
PGF2a	10.2	353.2	193.1			Negative	26
8-iso-13,14-dihydro-15-keto-PGF2a	10.6	353.2	183.1			Negative	27
LXA5	10.5	349.2	115.1			Negative	15
8-iso-PGE2	10.6	351.2	271.2			Negative	17
PGE2-d4	10.7	355.2	275.2			Negative	19
PGE2	10.6	351.2	271.2			Negative	17
11-dehydro-TXB2	10.9	367.2	305.2			Negative	17
8-iso-PGE1	10.9	353.2	235.2			Negative	17
15-keto-PGF2a	10.8	351.2	219.2			Negative	17
5S,14R-LXB4	11.3	351.3	221.2			Negative	15
PGK2	11.4	349.2	249.2	349.2	287.2	Negative	15
PGE1	11.0	353.2	235.2			Negative	16
PGD2-d4	11.0	355.2	275.2			Negative	18
PGD2	11.1	351.2	271.2	351.2	233.2	Negative	18
PGD1	11.1	353.2	235.1			Negative	14
11-beta-13,14-dihydro-15-keto-PGF2a	11.4	353.2	183.1			Negative	25
15-keto-PGE2	11.3	349.2	113.1			Negative	21
Resolvin D2	11.6	375.2	175.1	375.2	141.1	Negative	24
14,15-LTC4	11.8	626.4	308.2			Positive	-17
13,14-dihydro-15-keto-PGF2a	11.7	353.2	113.1			Negative	28
5S,6R-LXA4	11.8	351.2	115.1			Negative	15
13,14-dihydro-15-keto-PGE2	11.9	351.2	175.1			Negative	24
Resolvin D1	12.0	375.2	141.1			Negative	15
LTD4	12.3	497.3	189.1			Positive	-19
5S,6S-LXA4	12.4	351.2	115.1			Negative	15
14,15-LTE4	12.5	440.3	301.2			Positive	-13
1a1b-dihomo-PGF2a	12.5	381.2	337.2			Negative	23
13,14-dihydro-15-keto-PGD2	12.5	351.2	207.2			Negative	19
LTB4-EA	12.7	362.2	189.1			Positive	-16
LTC4-d5	12.8	631.4	308.2			Positive	-16
LTC4	12.8	626.4	308.2			Positive	-17
LTE4	12.9	440.3	189.1			Positive	-15
LTF4	12.9	569.4	251.2			Positive	-16
8-iso-PGA2	12.8	333.2	271.2			Negative	20
11-trans-LTC4	12.7	626.4	189.1			Positive	-21
8-iso-PGA1	13.0	335.2	235.2			Negative	13
11-trans-LTE4	13.1	440.3	189.1			Positive	-16
PGA2	13.0	333.2	271.2			Negative	15
PGJ2	13.1	333.2	271.2			Negative	15
PGB2	13.2	333.2	175.1			Negative	19
PGA1	13.4	335.2	235.2			Negative	14
8,12-iso-iPF2a-VI-1,5-lactone	13.9	337.2	265.2			Positive	-12
8,15-DiHETE	13.8	335.2	127.1			Negative	21
17,18-DiHETE	13.8	335.2	247.2			Negative	17
6-trans-LTB4	14.0	335.2	195.1			Negative	14
5,15-DiHETE	14.0	335.2	173.1			Negative	15
Maresin1	14.0	359.2	113.1	359.2	113.1	Negative	18
LTB4 d4	14.1	339.2	197.1			Negative	16
LTB4	14.1	335.2	195.1			Negative	15
10,17-DiHDoHE	14.2	359.2	153.1			Negative	17
13,14-dihydro-15-keto PGJ2	14.2	333.2	175.1			Negative	18

Compoubds	Retention time (min)	SRM		SRM (validation)		Polarity	Collision energy (eV)
		Q1	Q3	Q1	Q3		
13,14-dihydro-15-keto PGJ2	14.2	333.2	175.1			Negative	18
14,15-DiHETE	14.3	335.2	207.2			Negative	17
7,17-hydroxy-DPA	14.4	361.2	143.1	361.2	199.2	Negative	16
12,13-DiHOME	14.5	313.2	183.1			Negative	23
9,10-DiHOME	14.7	313.2	201.2			Negative	22
12-keto-LTB4	14.6	333.2	179.1			Negative	13
5,6-DiHETE	14.8	335.2	145.1			Negative	19
N-acetyl-LTE4	14.9	480.3	333.2			Negative	20
14,15-DHET	15.1	337.2	207.2			Negative	17
12-HHT	15.2	279.2	179.1			Negative	11
11,12-DHET	15.4	337.2	167.1			Negative	19
8,9-DHET	15.6	337.2	127.1			Negative	22
9-HOTrE	15.7	293.2	171.1			Negative	15
20-carboxy-AA	15.8	333.2	297.2	333.2	289.2	Negative	20
14,15-EET-EA	15.6	364.2	62.1			Positive	-18
5,6-DHET	15.9	337.2	145.1			Negative	18
13-HOTrE	15.9	293.2	195.1			Negative	17
18-HEPE	16.0	317.2	215.2			Negative	13
19-HETE	16.9	319.2	275.2			Negative	15
15-deoxy-delta-12,14-PGJ2	16.9	315.2	271.2			Negative	13
20-HETE	16.0	319.2	245.3			Negative	17
11,12-EET-EA	16.1	364.2	62.1			Positive	-22
15-HEPE	16.2	317.2	219.2			Negative	12
18-HETE	16.3	319.2	261.2			Negative	14
8,9-EET-EA	16.4	364.2	62.1			Positive	-22
17-HETE	16.5	319.2	247.2			Negative	15
12-HEPE	16.5	317.2	179.1			Negative	14
5,6-EET-EA	16.6	364.2	62.1			Positive	-23
16-HETE	16.5	319.2	233.2			Negative	13
5-HEPE	16.5	317.2	115.1			Negative	14
Lyso-PAF	16.4	482.3	184.1	482.3	184.1	Positive	-29
15-HpEPE	16.7	333.2	111.1			Negative	10
13-HODE	16.6	295.2	195.1			Negative	18
9-HODE	16.9	295.2	171.1			Negative	18
12-HpEPE	16.8	333.2	271.2			Negative	8
20-HDoHE	16.9	343.2	241.2			Negative	13
15-HETE-d8	17.0	327.2	226.2			Negative	14
5-HpEPE	16.8	333.2	173.1			Negative	10
15-HETE	17.0	319.2	219.2			Negative	13
9-HpODE	17.1	311.2	185.2			Negative	15
13-KODE	17.0	293.2	113.1			Negative	22
13-HpODE	17.1	311.2	113.1			Negative	16
16-HDoHE	17.1	343.2	233.2			Negative	14
17-HDoHE	17.1	343.2	245.2			Negative	12
9-KODE	17.3	293.2	185.1			Negative	20
11-HETE	17.1	319.2	167.1			Negative	16
13-HDoHE	17.2	343.2	193.1			Negative	13
10-HDoHE	17.2	343.2	153.1			Negative	15
8-HETE	17.3	319.2	155.1			Negative	14
12-HETE-d8	17.3	327.2	184.3			Negative	15
14-HDoHE	17.3	343.2	205.2			Negative	12
15-HpETE	17.4	335.2	113.1			Negative	10
12-HETE	17.3	319.2	179.1			Negative	14
11-HDoHE	17.4	343.2	149.1	343.2	121.1	Negative	13
7-HDoHE	17.4	343.2	141.1			Negative	13
9-HETE	17.5	319.2	123.1			Negative	17
5-HETE-d8	17.4	327.2	116.1			Negative	15
8-HDoHE	17.4	343.2	109.1			Negative	14
5-HETE	17.4	319.2	115.1			Negative	14
15-HETrE	17.5	321.2	221.2	321.2	113.1	Negative	15
PAF-d4	17.5	572.4	59.1			Negative	35
PAF	17.5	568.4	59.1			Negative	31
12-HpETE	17.6	335.2	153.1			Negative	10
12-KETE	17.6	317.2	153.1			Negative	16
5,6-DHET-lactone	17.8	321.2	177.1			Positive	-13
4-HDoHE	17.7	343.2	101.1			Negative	14
5-HpETE	17.9	335.2	129.1			Negative	11
12,13-EpOME	18.0	295.2	195.2			Negative	16
9,10-EpOME	18.1	295.2	171.1			Negative	16
14,15-EET	17.9	319.2	113.1			Negative	18
5-KETE	18.2	317.2	203.2			Negative	18
Azelaoyl-PAF	18.1	650.4	201.2			Negative	31
15-HEDE	18.2	323.2	223.2			Negative	19
AEA	18.3	348.2	62.1			Positive	-15
11,12-EET	18.4	319.2	167.1			Negative	13
8,9-EET	18.5	319.2	127.1			Negative	16
5,6-EET	18.5	319.2	191.1			Negative	10
15-KEDE	18.8	323.2	207.2			Positive	-14
OEA-d4	19.2	330.2	66.1			Positive	-19
OEA	19.2	326.2	62.1			Positive	-18
EPA-d5	19.5	306.2	262.2			Negative	11
EPA	19.5	301.2	257.2			Negative	10
DHA-d5	20.4	332.2	288.2			Negative	11
DHA	20.3	327.2	283.2			Negative	11
AA-d8	20.5	311.2	311.2	311.2	267.2	Negative	7
AA	20.5	303.2	303.2	303.2	259.2	Negative	12
