# Supplementary material for: Prostaglandin in the ventromedial hypothalamus regulates peripheral glucose metabolism
Source: Nat Commun. 2021 Apr 20;12:2330. doi: 10.1038/s41467-021-22431-6 (PMC8058102; doi:10.1038/s41467-021-22431-6)
Supplement: Supplementary file 3 — Reporting Summary [file 41467_2021_22431_MOESM3_ESM.pdf]

nature research

Corresponding author(s): Chitoku Toda

Last updated by author(s): Jan 25, 2021

Reporting Summary

Nature Research wishes to improve the reproducibility of the work that we publish. This form provides structure for consistency and transparency in reporting. For further information on Nature Research policies, see our Editorial Policies and the Editorial Policy Checklist.

Statistics

For all statistical analyses, confirm that the following items are present in the figure legend, table legend, main text, or Methods section.

n/a	Confirmed	
☐	☒	The exact sample size (*n*) for each experimental group/condition, given as a discrete number and unit of measurement
☐	☒	A statement on whether measurements were taken from distinct samples or whether the same sample was measured repeatedly
☐	☒	The statistical test(s) used AND whether they are one- or two-sided<br>*Only common tests should be described solely by name; describe more complex techniques in the Methods section.*
☒	☐	A description of all covariates tested
☒	☐	A description of any assumptions or corrections, such as tests of normality and adjustment for multiple comparisons
☐	☒	A full description of the statistical parameters including central tendency (e.g. means) or other basic estimates (e.g. regression coefficient) AND variation (e.g. standard deviation) or associated estimates of uncertainty (e.g. confidence intervals)
☐	☒	For null hypothesis testing, the test statistic (e.g. *F*, *t*, *r*) with confidence intervals, effect sizes, degrees of freedom and *P* value noted<br>*Give P values as exact values whenever suitable.*
☒	☐	For Bayesian analysis, information on the choice of priors and Markov chain Monte Carlo settings
☒	☐	For hierarchical and complex designs, identification of the appropriate level for tests and full reporting of outcomes
☒	☐	Estimates of effect sizes (e.g. Cohen's *d*, Pearson's *r*), indicating how they were calculated

Our web collection on statistics for biologists contains articles on many of the points above.

Software and code

Policy information about availability of computer code

Data collection	flexImaging 4.1
Data analysis	GraphPad Prism 9.0.0, ImageJ 1.53c

For manuscripts utilizing custom algorithms or software that are central to the research but not yet described in published literature, software must be made available to editors and reviewers. We strongly encourage code deposition in a community repository (e.g. GitHub). See the Nature Research guidelines for submitting code & software for further information.

Data

Policy information about availability of data

All manuscripts must include a data availability statement. This statement should provide the following information, where applicable:

- Accession codes, unique identifiers, or web links for publicly available datasets
- A list of figures that have associated raw data
- A description of any restrictions on data availability

The data that support the findings of this study are available in the source data files. The other data that supports the findings of this study are available from the corresponding author upon reasonable request. Source data are provided with this paper.

Field-specific reporting

Life sciences study design

All studies must disclose on these points even when the disclosure is negative.

Sample size	Sample size was selected based on previous experiences and standards in the field (Toda C, Cell, 2016; Toda C, Diabetes, 2013; Toda C, Diabetes, 2009). Sample size was also chosen to ensure an alpha value of 0.05.
Data exclusions	Data were not excluded from analysis.
Replication	All experiments described in this paper have been done more than twice and all attempts at replication were successful.
Randomization	All mice were randomly selected to inject inhibitors, chemicals or AAVs.
Blinding	In the hyperinsulinemic euglycemic clamp test, investigators were blinded to group allocation during data collection. We tried to perform blinding in all the other experiments, but it was not always possible. Thus, we changed researchers to replicate the experiment without telling them group allocation. All attempts at replication were successful independently on the researcher. As a result, we were blinded to group allocation in more than 70% of our entire experiments. We believe that the data was not biased.

Reporting for specific materials, systems and methods

We require information from authors about some types of materials, experimental systems and methods used in many studies. Here, indicate whether each material, system or method listed is relevant to your study. If you are not sure if a list item applies to your research, read the appropriate section before selecting a response.

Materials & experimental systems

n/a	Involved in the study	
☐	☒	Antibodies
☒	☐	Eukaryotic cell lines
☒	☐	Palaeontology and archaeology
☐	☒	Animals and other organisms
☒	☐	Human research participants
☒	☐	Clinical data
☒	☐	Dual use research of concern

Methods

n/a	Involved in the study	
☒	☐	ChIP-seq
☒	☐	Flow cytometry
☒	☐	MRI-based neuroimaging

Antibodies

Antibodies used	All the information of antibodies used in this study were described in the method and Supplemental table 1
Validation	All the antibodies were validated by each manufacturer. Antibodies used in this study are also used in many papers published by other researchers.<br>Rabbit-anti-cFos, Santa Cruz, SC-52, reactivity: included mouse, application: included immunohistochemistry (Magno et al., J Neurosci. 39(7):3234-3248, 2019).<br>Rabbit-anti-Iba1, FUJIFILM Wako, LKN5648, reactivity: human, mouse and rat, application: immunohistochemistry. https://labchem-wako.fujifilm.com/us/product/detail/W01W0101-1974.html<br>Rabbit-anti-GFAP, Sigma-Aldrich, HPA056030, reactivity: human and mouse, application: immunofluorescence and immunohistochemistry. https://www.sigmaaldrich.com/catalog/product/sigma/hpa056030?lang=en&region=CA<br>Rabbit-anti-GFP, Frontier institute, AB_2571573, reactivity: mouse, application: immunohistochemistry. https://nittobo-nmd.co.jp/pdf/reagents/GFP.pdf<br>Rabbit-anti-pSTAT3 (Tyr 705) antibody, Cell Signaling Technologies, 9145S, reactivity: included human, rat, mouse, and monkey. Application: included western blot and immunohistochemistry. https://www.cellsignal.com/products/primary-antibodies/phospho-stat3-tyr705-d3a7-xp-rabbit-mab/9145<br>Phospho-cPLA2 (Ser505) Antibody, Cell Signaling Technologies, 2831S, reactivity: included human, rat, mouse, and monkey, application: western blot and immunoprecipitation. https://www.cellsignal.com/products/primary-antibodies/phospho-cpla2-ser505-antibody/2831<br>cPLA2 Antibody, Cell Signaling Technologies, 2832S, reactivity: included human, rat, mouse, and monkey, application: western blot and immunoprecipitation. https://www.cellsignal.com/products/primary-antibodies/cpla2-antibody/2832

Animals and other organisms

Policy information about studies involving animals; ARRIVE guidelines recommended for reporting animal research

Laboratory animals	Male C57BL/6J mice (Charles River Laboratories Japan): 8-10 weeks old animals were used for IMS, LC-MS, GTT, ITT, cannulation, AAV injection, startting HFD.

	Male and female Sf1-cre mice (Jackson Laboratory: STOCK Tg(Nr5a1-cre)7Lowl/J): 8-10 weeks old on the day of viral injection and starting HFD.
Wild animals	No wild animals were used in the study.
Field-collected samples	No field-collected samples were used in the study.
Ethics oversight	the Animal Care and Use Committee of Hokkaido University

Note that full information on the approval of the study protocol must also be provided in the manuscript.
